# Novel Anti-Inflammatory Bioactive Peptide Derived from Yak Bone Collagen Alleviates the Skin Inflammation of Mice by Inhibiting the NF-κB Signaling Pathway and Modulating Skin Microbiota

**DOI:** 10.3390/foods14244238

**Published:** 2025-12-10

**Authors:** Zitao Guo, Tao Shi, Pengfei Xu, Zijun Wang, Bo Hu, Yu Xin, Zhongpeng Guo, Zhenghua Gu, Dake Dong, Liang Zhang

**Affiliations:** 1 School of Food and Biological Engineering, Jiangsu University, Zhenjiang 212013, China; guozt@ujs.edu.cn (Z.G.);; 2 National Engineering Research Center of Cereal Fermentation and Food Biomanufacturing, School of Biotechnology, Jiangnan University, Wuxi 214122, China; 3 National Engineering Research Center for Functional Food, Jiangnan University, Wuxi 214122, China; 4 Department of Dermatology, Affiliated Hospital of Jiangnan University, Wuxi 214122, China; 5 Engineering Research Center of the Ministry of Education for Wound Repair Technology, Jiangnan University, Affiliated Hospital of Jiangnan University, Wuxi 214000, China

**Keywords:** bioactive peptides, yak bone collagen, anti-inflammation, skin microbiota, NF-κB signaling pathway

## Abstract

Food-derived anti-inflammatory bioactive peptides have garnered significant attention due to their wide sources, easy absorption, and high biosafety. Our preliminary study identified a novel anti-inflammatory peptide GPAGPSGPAGKDGR (GR14) from yak bone collagen, which significantly alleviates lipopolysaccharide-induced inflammatory responses in HaCaT cells, demonstrating potential applications in improving skin inflammation. The objective of this study is to validate the anti-inflammatory effects of GR14 at the skin level in living animals and elucidate its mechanism of action. In this study, a 1-chloro-2,4-dinitrobenzene (DNCB)-induced skin inflammation mouse model was established. Results indicated that the GR14 intervention significantly improved skin manifestations and serum inflammatory cytokine levels in model mice. Compared to the model group, the high-dose treatment group demonstrated 33.61%, 40.55%, 22.18%, and 31.25% reductions in interleukin-6, interleukin-1β, tumor necrosis factor-α, and nitric oxide levels, respectively. Western blotting confirmed that GR14 intervention significantly inhibited the phosphorylation levels of P65 and IκB in the NF-κB signaling pathway. Microbiota analysis revealed that GR14 intervention effectively restored the significant decreases in *Staphylococcus* and *Mammaliicoccus* abundances caused by DNCB stimulation, returning the skin microbiota structure to normal status. These findings suggested that GR14 alleviates skin inflammation in model mice, potentially through suppressing the NF-κB signaling pathway and reshaping the skin microbiota structure. This study would promote the application of GR14 as a bioactive ingredient in the fields of functional food and functional cosmetics.

## 1. Introduction

Inflammation is an immune stress response that serves as the body’s self-defense mechanism against various external or endogenous stimuli [1]. This response has been implicated in the pathogenesis of multiple diseases. In the context of metabolic systems, inflammatory processes trigger a dramatic surge in macrophage infiltration and inflammatory cytokine levels within adipose tissue, ultimately contributing to obesity and diabetes mellitus [2]. Regarding neurological systems, excessive pro-inflammatory cytokines released during inflammatory reactions induce neuronal damage, which may lead to neurodegenerative disorders such as Alzheimer’s disease and Parkinson’s disease [3]. Within digestive systems, inflammatory-mediated intestinal mucosal damage may progress to Crohn’s disease and ulcerative colitis depending on the extent of affected tissue areas [4]. Furthermore, emerging evidence indicates that chronic inflammation induced by persistent infections and metabolic dysregulation is associated with increased cancer risk and accelerated tumor progression. Conventional therapeutic strategies for inflammation typically employ non-steroidal anti-inflammatory drugs (NSAIDs), glucocorticoids, or antibiotics; however, these agents are associated with significant adverse effects [5]. Although novel biologic agents such as monoclonal antibodies can precisely target and inhibit inflammatory pathways, their prohibitively high cost and the need for further validation of long-term safety and sustained efficacy remain significant concerns [6]. Consequently, identifying cost-effective and safe anti-inflammatory agents remains a central challenge.

Bioactive peptides, typically composed of 2–20 amino acids with low molecular weights for enhanced absorption, exhibit diverse biological activities including antioxidant, antimicrobial, and immunomodulatory properties [7]. Exogenous bioactive peptides are primarily obtained through enzymatic hydrolysis of dietary proteins, offering the advantages of broad source availability and high safety profiles [8]. Currently, the application of anti-inflammatory bioactive peptides has emerged as a prominent therapeutic strategy in inflammatory disease management [9]. Studies have identified anti-inflammatory effects of various mammalian collagens. For instance, porcine collagen hydrolysates could alleviate renal anemia in the chronic kidney disease mouse model through the anti-inflammatory protection. Compared with the model group, the intervention of porcine collagen hydrolysates reduced about 30% in the concentration of proinflammatory markers in serum and kidney [10]. Moreover, it has been reported that peptides derived from bovine bone collagen not only inhibit the extra secretion of proinflammatory cytokines in lipopolysaccharide (LPS)-induced RAW264.7 cells but also demonstrate their anti-inflammatory effects in dextran sodium sulfate (DSS)-induced C57BL/6 mice [11]. These findings collectively indicate that the mammalian collagen is a good resource to produce the anti-inflammatory peptides.

Yaks (*Bos grunniens*), primarily distributed in cold regions above 3000 m on the Qinghai-Tibet Plateau, exhibit remarkable cold resistance and extreme environmental adaptability, serving as a vital economic livestock species for pastoral communities in high-altitude areas [12]. Notably, yak bone collagen is rich in bioactive components, with enzymatic hydrolysates demonstrating multifunctional properties including antioxidant, antihypertensive, antitumor, cryoprotective, and lipid metabolism-regulating activities [13]. In our previous investigation, an anti-inflammatory peptide GR14 (GPAGPSGPAGKDGR) was separated, purified, and identified from yak bone collagen hydrolysates by employing ultrafiltration, reverse-phase high-performance liquid chromatography (RP-HPLC), and nano-liquid chromatography–tandem mass spectrometry (nano LC-MS/MS). Moreover, the anti-inflammatory effects of it were verified by using an LPS-induced HaCaT inflammatory cell model [12,14]. However, these findings were derived from in vitro studies, necessitating further validation of their therapeutic efficacy and detailed mechanism exploration in vivo. In this study, a skin inflammation mouse model was established by topical application of 1-chloro-2,4-dinitrobenzene (DNCB) to mice. The potential of GR14 against skin inflammation was investigated through a comprehensive evaluation of skin physiological parameters, including barrier function restoration and inflammatory response mitigation. Furthermore, the anti-inflammatory mechanism of GR14 was revealed. The findings of this study would further highlight the anti-inflammatory effects of GR14 and demonstrate it as a novel bioactive compound against skin inflammation, while concurrently creating a sustainable valorization strategy to transform yak-derived byproducts into high-value biomaterials.

## 2. Materials and Methods

### 2.1. Reagents

The peptide was synthesized by Shanghai Nouyou Biotechnology Co., Ltd. (Shanghai, China) with 95% purity as determined by HPLC. The protein concentration assay kit was purchased from Beyotime Biotechnology Co., Ltd. (Shanghai, China). The interleukin-6 (IL-6), interleukin-1β (IL-1β), tumor necrosis factor-α (TNF-α), nitric oxide (NO) assay kit was acquired from Nanjing Senbeijia Biotechnology Co., Ltd. (Nanjing, China). The antibodies were purchased from cell signaling technology (CST, Boston, MA, USA). Unless stated, all other chemicals were obtained from Sinopharm Chemical Reagent Co., Ltd. (Shanghai, China), with a chemical purity grade.

### 2.2. Preparation of Peptide-Containing Hydrogels

To ensure the intervention effect of GR14, the peptide was made into a hydrogel and applied to the modelling site. The blank hydrogel matrix was fabricated following Fan’s methodology, comprising (*w*/*w*): 1% Carbomer 90, 5% glycerol, 0.2% polysorbate 80, 5% dehydrated ethanol, and 0.05% ethylparaben as preservative [15]. After precise gravimetric measurement, excipients were solubilized in purified water under magnetic stirring (500 rpm, 25 °C), with subsequent pH adjustment to 6.5 ± 0.1 via triethanolamine titration. The compound dexamethasone acetate gel (positive drug gel) was prepared by thoroughly mixing compound dexamethasone acetate cream with blank gel at a 1:6.5 ratio. Subsequently, aqueous peptide solutions of varying concentrations are homogeneously mixed with the blank gel to achieve final peptide concentrations of 1 mg/g, 0.5 mg/g, and 0.1 mg/g, designated as high-, medium-, and low-concentration intervention formulations, respectively.

### 2.3. Animals and Experiment Design

The experiment utilized forty-eight 8-week-old male ICR mice (SPF grade, body weight around 35 g) obtained from Beijing Vital River Laboratory Animal Technology Co., Ltd. (Beijing, China). Animals were housed in the SPF-grade barrier facility at the animal experiment center of Jiangnan University under controlled conditions: temperature 23 ± 2 °C, relative humidity 55 ± 10%, and 12 h light/dark cycles. After one week adaptation period, mice were randomly allocated into six experimental groups (n = 8 per group): blank control group (NC), model group (MC, DNCB treatment and not receive any GR14), dexamethasone acetate treatment group as positive group (PC), low-dose GR14 group (LD, 0.1 mg/g), medium-dose GR14 group (MD, 0.5 mg/g), and high-dose GR14 group (HD, 1 mg/g). All animals were maintained in groups of four per cage with ad libitum access to autoclaved feed and sterile water. All mice were raised according to the guidelines for animal experimentation of the experiment center of Jiangnan University, and the study was approved by the Experimentation Animal Ethics Committee of Jiangnan University (Protocol code: JN. No 20241030i1401217[561]).

On the day before the experiment, a 2 × 3 cm dorsal area was depilated using hair removal cream. On day 1, all groups except the NC group received 100 μL of 7% DNCB acetone solution (prepared in 4:1 acetone/olive oil) for sensitization, while the NC group received vehicle control (4:1 acetone/olive oil). From day 6, mice were challenged with 10 μL 0.5% DNCB solution on the right ear and dorsal area every 3 days (total 5 challenges on days 6, 9, 12, 15, 18). Starting from day 5, therapeutic interventions were administered twice daily (0.5 g/time, total 1.0 g/day) on the dorsal and ear regions: the NC and MC groups received blank gel, various peptide hydrogel doses were applied to the treatment groups, and the PC group received an equal weight of 0.1 mg/g dexamethasone gel. When challenge and treatment coincided, a 2 h interval was maintained between applications. The mice were fasted but allowed free access to water on the night before the end of the experiment. The experiment concluded on day 21.

### 2.4. Skin Moisture Content

After the experiment, the mice were anesthetized with isoflurane (induction concentration 3–4%, maintenance concentration 1–1.5%) and euthanized by cervical dislocation. Subsequently, the full-thickness skin from the experimental area on the back was rapidly and completely excised. Connective tissue and subcutaneous fat were removed, and a quarter of the skin was trimmed as a sample. The wet weight (M1) of the sample was measured. The sample was then dried in an 80 °C oven until a constant weight was achieved, and its dry weight (M0) was recorded. The skin moisture content was calculated using Formula (1):

(1) $$Skin\;moisture\;content\left(\%\right)=\frac{M_{1}-M_{0}}{M_{1}}\times 100\%$$

### 2.5. Ear Thickness and Weight

Prior to sensitization on day 1, baseline right ear thickness was measured in all mice using a digital thickness gauge (accuracy 0.001 mm). Subsequently, ear thickness measurements were repeated every 3 days throughout the experimental period. Following termination of the experiment, bilateral ear tissues were collected during dissection using a 6 mm biopsy punch. The excised ear specimens were immediately weighed using an analytical balance (±0.1 mg sensitivity) and data were recorded.

### 2.6. Histopathological Analysis of Mouse Skin and Ear

The skin tissues collected from the dorsal region and the ear of mice were cleaned and immersed in 4% paraformaldehyde solution for 24 h fixation. Subsequently, hematoxylin-eosin (H&E) staining was performed according to a previously described method [16]. The epidermal thickness of the back skin and ear was calculated based on the results of the staining by employing ImageJ2.

### 2.7. Determination of Spleen Index

Prior to euthanasia via exsanguination, individual mouse body weights were recorded using a precision balance (±0.1 g). Following cervical dislocation, spleens were aseptically excised during necropsy and placed on pre-weighed filter paper. Splenic tissue was subsequently weighed using an analytical balance (±0.01 g sensitivity). The spleen index was calculated post-dissection using the standardized Formula (2) [17]:

(2) $$Spleen\;index\left(\%\right)=\frac{Spleen\;weight(mg)}{Body\;weight(g)}\times 100\%$$

### 2.8. Quantification of Serum Inflammatory Factors

Blood samples were collected via retro-orbital plexus puncture under isoflurane anesthesia. Whole blood was allowed to clot at room temperature for 60 min, followed by centrifugation at 2000× *g* for 15 min at 4 °C. Serum aliquots were aspirated using sterile low-retention pipette tips and stored at −80 °C until analysis. The concentrations of IL-1β, IL-6, TNF-α, and NO were quantified using commercially available ELISA kits following the manufacturer’s protocols (Nanjing Jiangcheng Bioengineering Institute, Nanjing, China). Absorbance measurements were performed in technical duplicates using a microplate reader with wavelength settings as specified in each kit’s documentation.

### 2.9. Western Blotting

The expression of the key proteins in the NF-κB signaling pathway was tested as previously described with a slight modification [18]. Briefly, skin tissues harvested from mice were homogenized in lysis buffer containing phenylmethanesulfonyl fluoride (PMSF) and protease inhibitors to extract total proteins. Protein concentration was determined using the BCA assay. Protein sample (20 μg) was separated by sodium dodecyl sulfate-polyacrylamide gel electrophoresis (SDS-PAGE), transferred onto PVDF membranes, and subjected to blocking and washing. The membranes were incubated overnight at 4 °C with the following primary antibodies: β-actin (1:5000), P65 (1:1000), phosphorylation P65 (p-P65, 1:1000), IκB (1:1000) and phosphorylation IκB (p-IκB, 1:1000). After TBST washing, horseradish peroxidase (HRP)-conjugated secondary antibody (1:5000) was applied and incubated at room temperature for 2 h. Protein bands were visualized using enhanced chemiluminescence, and band intensity was quantified with ImageJ software.

### 2.10. 16S rRNA Gene Sequencing

The alteration of skin microbiota under the peptide intervention was analyzed by applying 16S rRNA gene sequencing. On the day of experiment termination, the back skin microbiota of four mice in the NC group and eight mice in the other groups were sampled prior to anesthesia. A saline-moistened cotton swab was firmly rubbed 40 times (10 strokes in each direction: up, down, left, right) across the dorsal skin surface. The swab was immediately placed into a sterile sample tube and stored at −80 °C within 30 min. Four samples from the NC group and four randomly selected samples from each of the other groups, a total of 24 samples, were tested for skin microbiome composition. DNA extraction, sequencing, and bioinformatic analysis were performed as previously described [19].

### 2.11. Statistical Analysis

The software SPSS 25.0 (SPSS Inc., Chicago, IL, USA) was employed to analyze all data. One-way ANOVA was performed to evaluate the significant differences in the phenotypes and the relative abundance of skin microbes between groups. Unless stated, the results were presented by mean ± SD. The significant difference was accepted at *p* < 0.05 and *p* < 0.01 was a highly significant difference.

## 3. Results and Discussion

### 3.1. Effects of Anti-Inflammatory Peptides on the Appearance of Mouse Skin

**Figure 1:**
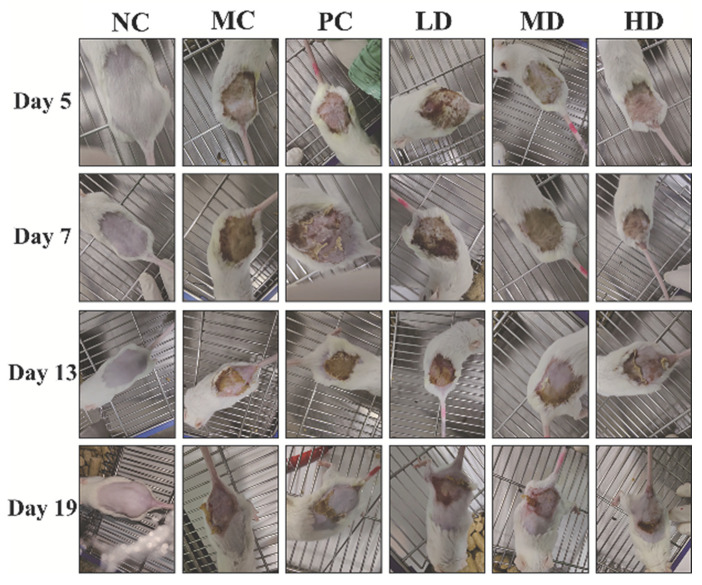
The changes in the skin of the same mouse in each group throughout the experiment. NC, blank control group; MC, model group; PC, dexamethasone acetate treatment group; LD, low-dose GR14 group; MD, medium-dose GR14 group; HD, high-dose GR14 group.

The DNCB-elicited murine recapitulates hallmark clinical manifestations, including erythematous papules, erosions, and epidermal desquamation in lesional skin areas—pathognomonic features consistent with human atopic dermatitis pathophysiology [20]. Figure 1 shows the apparent changes in the skin of the same mouse in each group after induction and during the low-dose challenge period. Following 7% DNCB-acetone sensitization on Day 5, all treatment groups except the NC group exhibited hallmark pathological features, including epidermal thickening, induration, and erythema. During the low-concentration DNCB challenge, progressive exacerbation of dorsal skin inflammation was observed in the MC group. When stimulated at a low concentration of DNCB, with the increase in the number of stimulations, the skin inflammation on the back of the mice in the model group began to become more severe, and the skin condition gradually developed from epidermal thickening to skin erosion after epidermal exfoliation, indicating that the atopic dermatitis model was successfully created. In contrast to the MC group, the PC group demonstrated rapid clinical improvement from the first treatment administration. By Day 19 (24 h post-final challenge), near-complete resolution of erythema was achieved in PC mice, with residual crusted areas exhibiting advanced re-epithelialization. Although the LD group showed comparable pathology to the MC group, both the MD and HD groups displayed dose-dependent therapeutic effects. Compared with the MC group, the MD group exhibited a significant reduction in erosion area, while the HD group achieved near-total resolution of erythema and erosions, demonstrating efficacy comparable to the PC group. In summary, these macroscopic observations collectively demonstrate that GR14 exhibits therapeutic efficacy in alleviating DNCB-induced cutaneous pathology.

### 3.2. Immune Response and Skin Moisture Content in Mice

**Figure 2:**
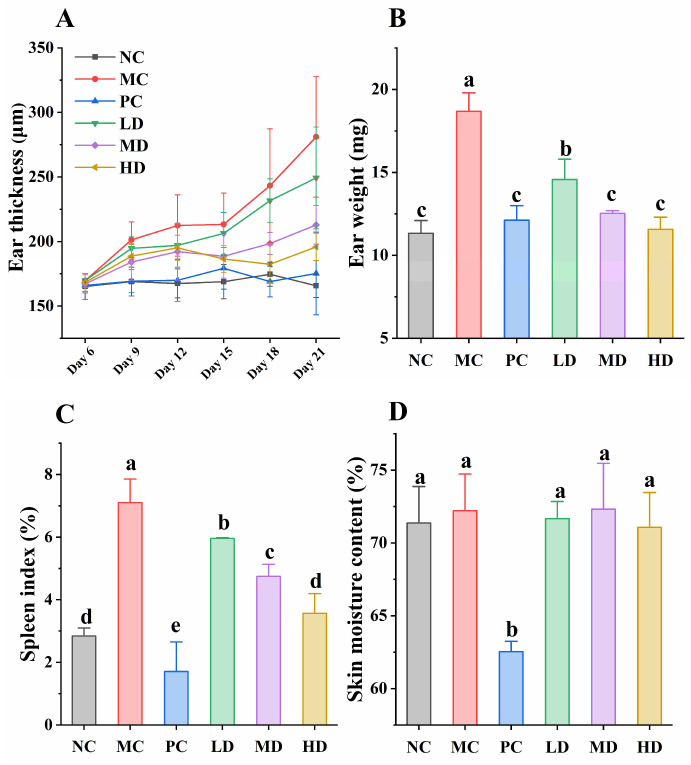
Effects of GR14 on the immune response and skin moisture content in mice. (**A**) The ear thickness of mice during intervention; (**B**) the ear weight of mice at the endpoint of intervention; (**C**) the spleen index of mice at the endpoint of intervention; (**D**) the skin moisture content in mice at the endpoint of intervention. NC, blank control group; MC, model group; PC, dexamethasone acetate treatment group; LD, low-dose GR14 group; MD, medium-dose GR14 group; HD, high-dose GR14 group. Different letters indicate the significant differences between groups (n = 8 per group, *p* < 0.05).

When the ears of mice were activated by applying a low-concentration DNCB solution to the mice, the ears of the mice also showed inflammation and swelling [21]. The change of ear thickness with excitation time in mice is shown in Figure 2A. There were no differences between groups at the beginning of the activation. However, the MC group exhibited time-dependent aggravation of auricular swelling, reaching 281.14 ± 46.77 μm by Day 21, which was significantly elevated compared to the NC group (165.75 ± 9.02 μm, *p* < 0.01). Compared to the NC group, the PC group showed minor fluctuations in ear thickness changes, but overall maintained relatively stable thickness. Notably, GR14 administration demonstrated dose-dependent efficacy in mitigating edema progression. Specifically, by day 21, the HD group exhibited an ear thickness of 195.86 ± 10.53 μm. Although this exceeded the PC group’s post-treatment thickness of 175.29 ± 31.99 μm, it demonstrated significant improvement compared to the MC group (*p* < 0.01). Additionally, ear weight intuitively reflected inflammatory status across groups. As shown in Figure 2B, compared to the NC group, the MC and LD groups showed significant increases in ear weight (*p* < 0.01), while the PC, MD, and HD groups showed no significant changes (*p* > 0.05), indicating that the anti-inflammatory peptide alleviated ear weight gain. These results visually demonstrate the improvement effects of GR14 on murine auricular inflammation.

Organ indices can reflect the related functions of the body and serve as biomarkers of disease status. The spleen, as a vital immune organ, is influenced by inflammation, leading to corresponding changes in spleen index [22]. It has been reported that DNCB-induced AD-like models typically cause splenomegaly and elevate spleen index in mice [15]. In this study, under DNCB stimulation, the MC group showed a significant increase in spleen index (Figure 2C, *p* < 0.01). In contrast, the PC group exhibited a notable decrease in spleen index, even lower than the NC group (*p* < 0.05). Dexamethasone inhibits the proliferation and activity of immune cells, significantly alleviating DNCB-induced inflammatory responses. However, as a glucocorticoid, it also might induce spleen atrophy to reduce spleen weight [23]. In GR14 intervention groups, spleen index was inversely proportional to treatment dosage, with the HD group showing no significant difference from the NC group (*p* > 0.05). These results demonstrate that GR14 can ameliorate splenomegaly in atopic dermatitis mouse models.

Skin moisture content is an important indicator for evaluating skin health status [24]. According to the results, except for the PC group, the skin hydration levels of all experimental groups ranged between 71–73% without significant differences (Figure 2D, *p* > 0.05). This might be related to the administration method, as the hydrogel carrier effectively maintained skin hydration, ensuring relatively consistent levels across these groups. However, the reduced skin hydration in the PC group likely occurred because glucocorticoids like dexamethasone inhibit lipid synthesis in keratinocytes, particularly the production of ceramides, cholesterol, and free fatty acids [25]. These lipids are crucial components of the skin barrier, and their reduction compromises barrier function, leading to increased water loss. This suggests that, compared to dexamethasone, the anti-inflammatory peptide does not cause significant damage to the skin barrier.

### 3.3. Histopathology of the Back Skin and the Ear

**Figure 3:**
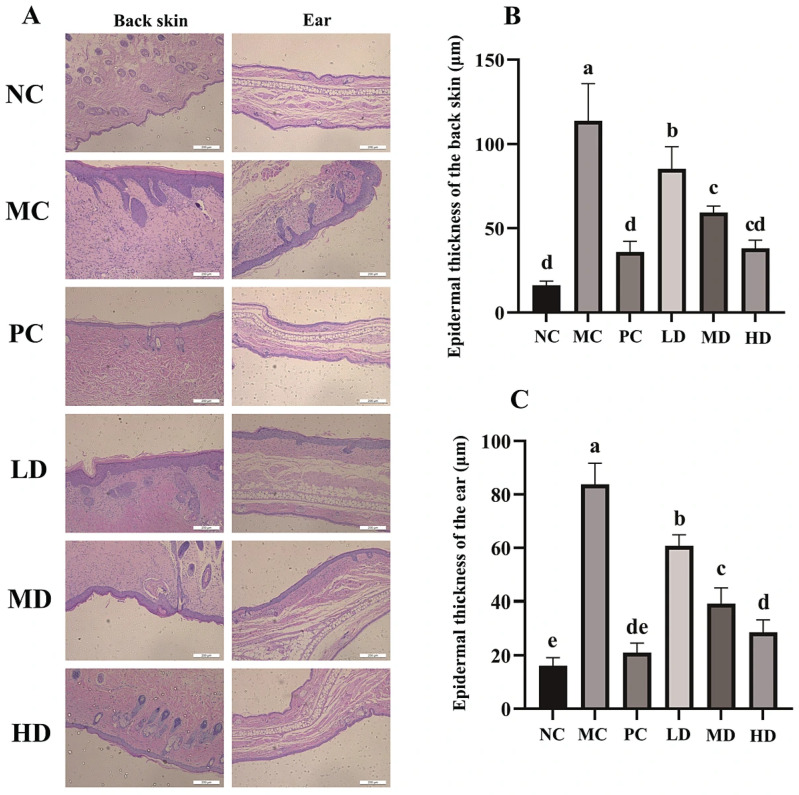
Histopathological analysis of skin on the back and ear of mice. (**A**) The H&E staining of skin in the back and ear of mice; (**B**) the epidermal thickness of the back skin; (**C**) the epidermal thickness of the ear. Different letters indicate the significant differences between groups (n = 8 per group, *p* < 0.05). NC, blank control group; MC, model group; PC, dexamethasone acetate treatment group; LD, low-dose GR14 group; MD, medium-dose GR14 group; HD, high-dose GR14 group. The scale bars were 200 μm and the magnification was 100 times in the histopathological pictures.

In addition to directly observing skin function through superficial appearance, deeper evaluation can be conducted using histopathological sections. The results show that mice in the MC group exhibited significant epidermal thickening in both dorsal and ear skin, particularly with pronounced hypertrophy of the stratum spinosum compared to the NC group (Figure 3A, *p* < 0.01). The epidermal thickness of dorsal and ear skin reached 113.83 ± 17.98 μm and 83.80 ± 6.44 μm, respectively, significantly higher than that of the blank control group (16.25 ± 2.02 μm and 16.10 ± 2.49 μm, Figure 3B,C, *p* < 0.05). Furthermore, substantial inflammatory infiltration was observed in the MC group’s skin tissues (Figure 3A). In the PC group, epidermal thickening was markedly suppressed, with thickness closely resembling that of the NC group (*p* > 0.05). Although the anti-inflammatory peptide intervention groups still showed noticeable epidermal thickening in dorsal and auricular tissues, compared to the MC group, effective improvement was observed, and the ameliorative effect increased with higher peptide concentrations (Figure 3A, *p* < 0.05). Notably, in the HD group, the epidermal thicknesses of the dorsal and ear were 38.109 ± 3.964 μm and 28.60 ± 3.78 μm, respectively, approaching the therapeutic efficacy of the PC group (35.92 ± 5.23 μm and 21.02 ± 2.97 μm, Figure 3B,C). Moreover, inflammatory infiltration within skin tissues was significantly alleviated (Figure 3A, *p* < 0.05).

### 3.4. Determination of Serum Inflammatory Factors in Mice

**Figure 4:**
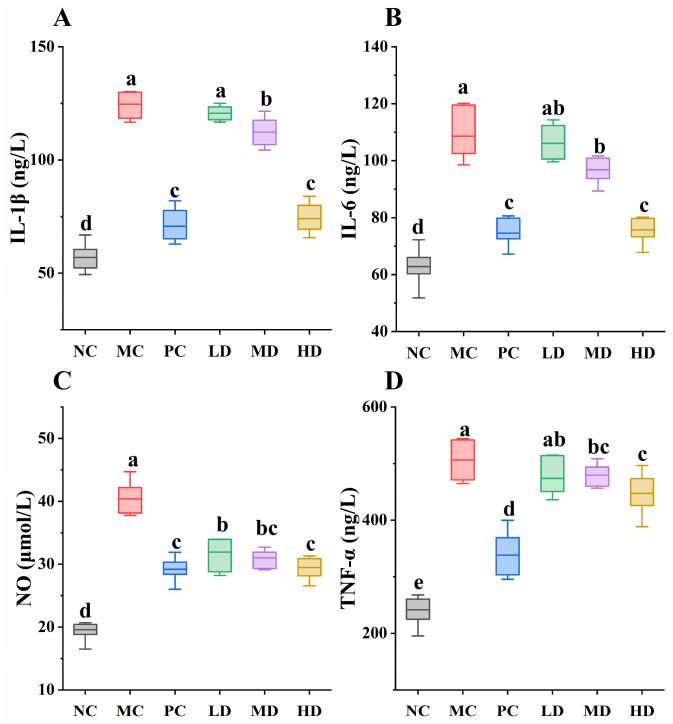
The concentration of inflammatory cytokines in the serum of mice. (**A**) IL-1β (interleukin-1β); (**B**) IL-6 (interleukin-6); (**C**) NO (nitric oxide); (**D**) TNF-α (tumor necrosis factor-α). NC, blank control group; MC, model group; PC, dexamethasone acetate treatment group; LD, low-dose GR14 group; MD, medium-dose GR14 group; HD, high-dose GR14 group. Different letters indicate the significant differences between groups (n = 8 per group, *p* < 0.05).

During the pathogenesis of skin disease, inflammation-associated signaling pathways are activated, leading to substantial release of inflammatory cytokines that drive inflammatory responses [26]. Therefore, evaluating the levels of inflammatory cytokines in serum serves as a reliable approach to assess the anti-inflammatory efficacy of bioactive peptides [27]. For instance, Zeng et al. demonstrated that the amphibian-derived anti-inflammatory peptide Esculentin-1GN alleviates murine inflammation by effectively reducing levels of four inflammatory mediators: IL-6, IL-1β, TNF-α, and NO [28]. Similarly, Ho et al. revealed that the corn silk-derived anti-inflammatory peptide FK2 exerts therapeutic effects through significant suppression of IL-1β in murine models [29]. In our previous study, the inhibition effects of GR14 on the secretion of inflammatory cytokines have been verified in the RAW 264.7 inflammatory cell model and HaCaT inflammatory cell model [12,14]. In the current study, the anti-inflammatory effect of GR14 was also observed. The serum concentrations of four inflammatory cytokines were markedly elevated in the MC group following DNCB stimulation, confirming successful model establishment (Figure 4). The PC group exhibited substantial reductions in all four cytokines. Notably, GR14-treated groups displayed a distinct dose-dependent attenuation of serum cytokine levels. The ameliorative effects became progressively pronounced with increasing peptide concentrations. There were 40.55%, 33.61%, 31.25%, and 22.18% reductions in the concentration of IL-1β, IL-6, NO, and TNF-α levels in the HD group when compared to the MC group. Remarkably, the concentrations of IL-1β, IL-6, and NO in the HD group showed no statistically significant differences from those in the PC group (Figure 4A–C, *p* > 0.05). The amino acid composition, hydrophobicity, and residue types at the C-terminal and N-terminal regions significantly influence the bioactivity of peptides [30]. Analysis of anti-inflammatory bioactive peptide sequences reveals that positively charged and hydrophobic amino acids at both N-terminal and C-terminal regions enhance their anti-inflammatory activity. In tuna juice and amaranth protein [31,32], peptides PRRTRMMNGGR and GPR were identified, both containing Arg at their C-terminus. In this study, GR14 also terminates with Arg at its C-terminal end. Hydrophobic amino acids contribute to anti-inflammatory efficacy, and GR14 contains two hydrophobic residues (proline and alanine), with proline recurring three times. The presence of hydrophobic amino acids facilitates peptide-membrane interactions, enabling bioactive peptides to modulate intracellular signaling pathways and exert anti-inflammatory functions. In summary, GR14 demonstrates remarkable efficacy in alleviating skin inflammation, where its hydrophobic amino acid content and C-terminal arginine residue likely play critical roles in mediating anti-inflammatory effects.

### 3.5. Effects of GR14 on the Phosphorylation of Key Proteins in the NF-κB Signaling Pathway

Upon exposure to inflammatory triggers, the body activates inflammatory signaling pathways, prompting immune cells to synthesize substantial quantities of cellular inflammatory factors [33]. These cytokines diffuse through paracrine or endocrine mechanisms, recruiting immune cells (e.g., neutrophils, macrophages) to inflammatory sites and amplifying inflammatory responses. Simultaneously, the released cytokines reciprocally modulate inflammatory pathways, establishing a mutually reinforcing cycle of inflammation [34]. Excessive inflammatory reactions may induce systemic discomfort or even tissue failure, making the suppression of inflammatory signaling pathways to mitigate cytokine overproduction a primary therapeutic strategy for inflammation control [35]. Research has identified three major inflammation-associated signaling pathways: the NF-κB pathway, mitogen-activated protein kinase (MAPK) pathway, and Janus kinase-signal transducer and activator of transcription (JAK-STAT) pathway [36,37]. Current studies indicate that the NF-κB pathway plays a more prominent role in inflammatory processes [38]. Functioning as a central inflammatory mediator, NF-κB responds to receptors involved in diverse immune responses. Pathway activation triggers IκB phosphorylation, leading to dissociation of the NF-κB-IκB complex and liberation of NF-κB dimers. These released dimers translocate into the nucleus, binding to target gene promoters to initiate transcription of inflammation-related genes [39]. Given that dysregulated NF-κB activation underlies various inflammatory diseases, targeted inhibition of this pathway represents a viable anti-inflammatory therapeutic approach. The growing success of NF-κB inhibitors in inflammation management underscores its pivotal role in inflammatory research [40,41]. In our previous investigation, GR14 demonstrated efficacy in reducing inflammatory cytokine levels in HaCaT keratinocyte models through NF-κB pathway suppression [14]. To further elucidate this mechanism, the current study employed Western blotting to quantify phosphorylation levels of key proteins within the NF-κB signaling cascade.

**Figure 5:**
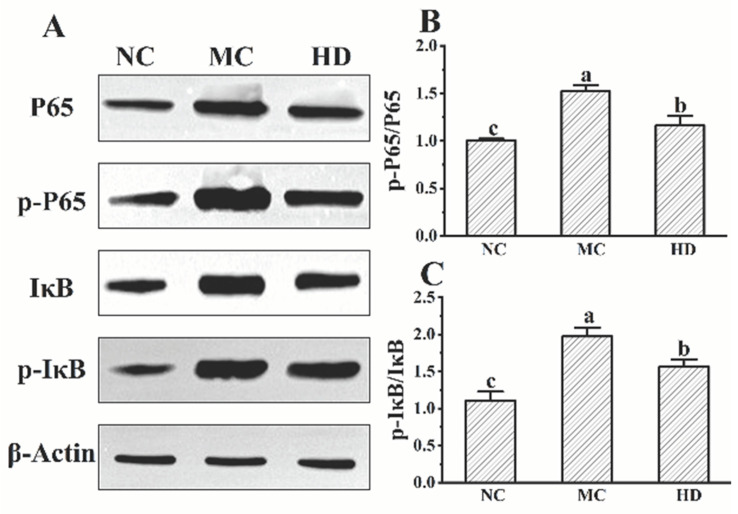
The Western blotting analysis of the phosphorylation levels of P65 and IκB in the NF-κB signaling pathway. (**A**) The gel of different groups; (**B**) the ratio of phosphorylation P65 (p-P65) to P65; (**C**) the ratio of phosphorylation IκB (p-IκB) to IκB. NC, blank control group; MC, model group; HD, high-dose GR14 group. Different letters indicate the significant differences between groups (n = 3 per group, *p* < 0.05). Note: the original gel was shown in Figure S1.

Based on physiological and biochemical analyses of skin tissues, high-dose GR14 treatment significantly alleviated inflammatory responses in mice. Accordingly, the Western blotting analysis primarily compared phosphorylation levels of key proteins in the NF-κB signaling pathway of the back skin of mice across the NC, MC, and HD groups (Figure 5A). Three skin samples from each group were randomly selected for detection. Results indicated that the phosphorylation of P65 and IκB in the MC group was more obvious than that in the NC and HD groups (*p* < 0.05). The ratio of p-P65/P65 and p-IκB/IκB in the MC group was separately elevated about 1.5 and 2.0 times higher than that in the NC group. After the intervention of high-dose GR14, the upward trend of phosphorylation was significantly inhibited (Figure 5B,C, *p* < 0.05). These results indicate that GR14 may exhibit anti-inflammatory effects by inhibiting the NF-κB signaling pathway.

### 3.6. Effects of GR14 on the Modulation of Skin Microbiota

As the primary interface between the human body and the external environment, the skin is colonized by a substantial microbial community [42]. When the composition, diversity, or functionality of this cutaneous microbiota undergoes dysregulation, the homeostatic equilibrium between commensal microorganisms and the host is disrupted, thereby triggering or exacerbating dermatological disorders [43]. It has been reported that skin microbiota plays a pivotal role in the pathogenesis of skin inflammation [42]. Therefore, evaluating GR14-induced modifications to skin microbial composition could elucidate novel mechanisms underlying its anti-inflammatory efficacy.

**Figure 6:**
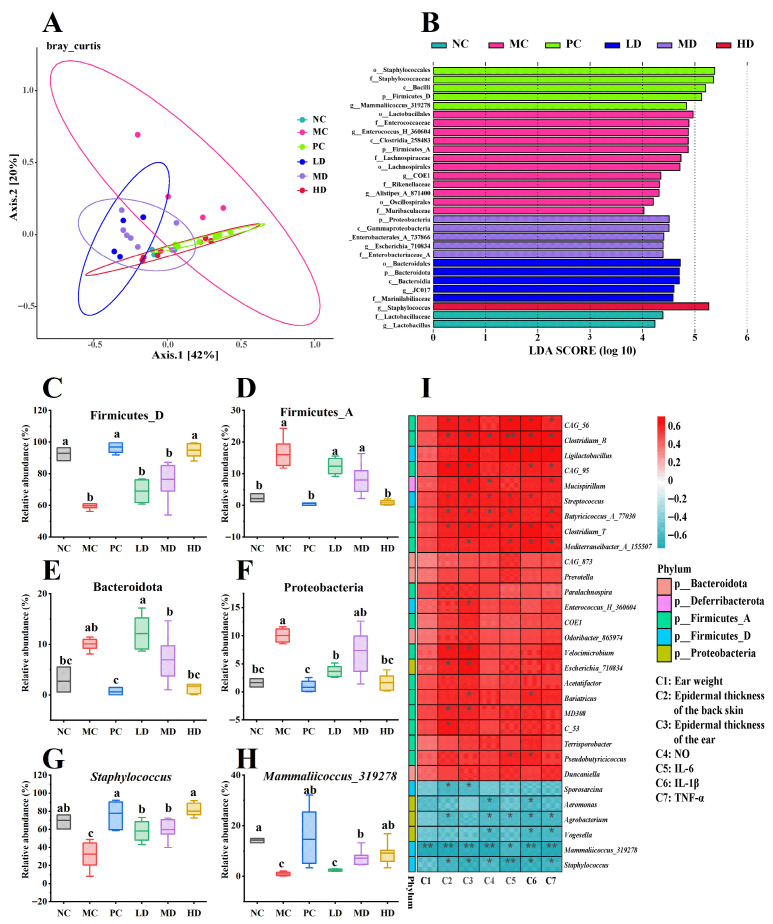
The skin microbiota analysis was performed by employing 16S rRNA gene sequencing. (**A**) The β-diversity of skin microbiota; (**B**) the LEfSe analysis of skin microbiota; (**C**–**H**) the relative abundance of Firmicutes_D, Firmicutes_A, Bacteroidota, Proteobacteria, Staphylococcus, and Mammaliicoccus_319278, respectively; (**I**) correlation analysis between skin microbiota and biomarkers of atopic dermatitis-like model mice. NC, blank control group; MC, model group; PC, dexamethasone acetate treatment group; LD, low-dose GR14 group; MD, medium-dose GR14 group; HD, high-dose GR14 group. Different letters indicate the significant differences between groups (n = 4 per group, *p* < 0.05). “*” indicates that there is a significant correlation between the two parameters, *p* < 0.05; “**”, indicates that there is a highly significant correlation between the two parameters, *p* < 0.01.

The Ace and Chao indices were employed to assess microbial abundance, while Shannon and Simpson indices were utilized to evaluate diversity within the cutaneous microbiota. As demonstrated by the results, no significant differences were observed in Ace and Chao indices across treatment groups compared to the NC group, indicating minimal variations in microbial load (Table 1, *p* > 0.05). Notably, the MC and LD groups exhibited significant increases in Shannon index relative to the NC group (*p* < 0.05), with the MC group specifically showing elevated Simpson index values (*p* < 0.01). Further β-diversity analysis via Principal Coordinates Analysis (PCoA) revealed that both the PC and HD groups clustered closely with the NC group, indicating minimal structural divergence in skin microbiota compared to pre-treatment states (Figure 6A). Notably, along Axis 2, the LD and MD groups exhibited closer proximity to the MC group, suggesting model-induced dysbiosis patterns. Collectively, the dermatitis induction protocol significantly altered microbiota architecture, whereas GR14 intervention demonstrated a positive restorative effect on community structure diversity.

The differential analysis of intergroup differences in the microbiota composition was performed using LEfSe (Linear discriminant analysis Effect Size). At the phylum level, four phyla, including Firmicutes_D, Firmicutes_A, Bacteroidota, and Proteobacteria, exhibited significant abundance variations across groups (Figure 6B and Figure S2, *p* < 0.05). In normal skin, Firmicutes is the primary microbial phylum that maintains the homeostasis of the skin barrier system. For instance, the Lactobacillus genus within this phylum can resist external stimuli [43]. In this study, the relative abundance of Firmicutes in the NC group was above 90% (Firmicutes_D accounted for 92.98 ± 4.21%, while Firmicutes_A accounted for 2.17 ± 1.32%). Thus, changes in the relative abundance of Firmicutes play a critical role in maintaining microbial structural homeostasis. Typically, Firmicutes_A is the dominant constituent of gut microbiota, whereas Firmicutes_D constitutes the primary component of skin microbiota [44], which is consistent with our results (Figure 6C,D). Compared to the NC group, the MC group exhibited a significant decrease in the relative abundance of Firmicutes_D (*p* < 0.05). This indicates that the establishment of the AD-like model significantly disrupted the homeostasis of skin microbiota. In healthy skin, Bacteroidota and Proteobacteria generally occupy low proportions, and an increase in their colonization abundance may lead to infections [45,46]. Consistent with this finding, in this study, the MD, LD, and MC groups showed significant increases in the proportions of Bacteroidota and Proteobacteria (*p* < 0.05), with the combined mean abundances of these two phyla reaching 14.33%, 15.78%, and 20.06%, respectively. This suggests impaired skin barrier function in these three experimental groups. In contrast, the phylum composition in the HD and PC groups showed no significant differences compared to the NC group (*p* > 0.05).

At the genus level, a total of eight genera showed significant differences (Figure 6B, *p* < 0.05). According to the analysis of the microbial community structure at the genus level (Figure S3), compared to the NC group, the MC group had significant differences primarily in *Staphylococcus*, *Mammaliicoccus_319278*, *Enterococcus_H_360604*, and *Alistipes_A_871400* (*p* < 0.05). The differences in the abundance of *Staphylococcus* and *Mammaliicoccus_319278* were statistically significant (*p* < 0.05). However, the abundance of *Enterococcus_H_360604*, and *Alistipes_A_871400* showed considerable variations among samples within the MC group, which were not statistically significant when compared to the NC group (*p* > 0.05). In normal healthy skin, the microbial genera on the skin surface are predominantly composed of *Staphylococcus*, which belongs to the phylum Firmicutes [2]. The representative species within *Staphylococcus* is *Staphylococcus epidermidis*, a resident and dominant species on the skin surface [47]. *S. epidermidis* inhibits the colonization of pathogenic bacteria such as *Staphylococcus aureus* and *Streptococcus pyogenes* by rapidly proliferating to occupy skin surface space and nutrients. Simultaneously, it forms protective biofilms to prevent pathogen adhesion and secretes phenol-soluble modulinos to lyse pathogen cell membranes [48]. In this study, the abundance of *Staphylococcus* in the MC group (32.72 ± 17.58%) was significantly lower than in other experimental groups (*p* < 0.05). The abundances of Staphylococcus in the LD and MD groups were 58.35 ± 13.02% and 59.71 ± 11.12%, respectively. Although these values showed significant improvement compared to the MC group, they remained significantly lower than the abundance in the NC group (70.04 ± 8.50%, *p* < 0.05). The Staphylococcus abundances in the PC and HD groups were 77.78 ± 14.18% and 80.20 ± 7.17%, respectively, showing no significant difference from the NC group and even a slight increase (*p* > 0.05). This suggests that the anti-inflammatory peptide effectively alleviates the inflammation-induced decline in *Staphylococcus* abundance and may promote its colonization on the skin surface. *Mammaliicoccus* is a recently reclassified genus from *Staphylococcus*, with limited research available [49]. Based on its original classification under *Staphylococcus*, it is hypothesized that *Mammaliicoccus* may share similar functions. The abundance of *Mammaliicoccus* in the MC group (0.96 ± 0.88%) was substantially reduced compared to other groups (Figure 6H, *p* < 0.05). As the GR14 intervention concentration increased, the abundance of *Mammaliicoccus* gradually rose. The relative abundance of *Mammaliicoccus* in the LD, MD, and HD groups was 2.47 ± 0.48%, 7.11 ± 3.02% and 9.18 ± 4.25%, respectively. Meanwhile, the *Mammaliicoccus* abundance in the PC group (14.34 ± 11.55%) was comparable to that in the blank control group (14.65 ± 0.93%, *p* > 0.05).

Microorganisms influence local and systemic immunity in the human body through their cellular components and metabolites acting on host cells [42]. To further investigate how skin microbiota mediates GR14’s improvement of immune function and inflammatory responses in model mice, we conducted a correlation analysis between skin microbiota and biomarkers of AD-like model mice (Figure 6I). At the phylum level, multiple genera within Firmicutes_A showed significant positive correlations with biomarkers, while three genera each from Firmicutes_D and Proteobacteria exhibited significant negative correlations (*p* < 0.05). At the genus level, *Staphylococcus* and *Mammaliicoccus* demonstrated significant negative correlations with skin thickness and inflammatory cytokines (*p* < 0.05). Although numerous genera showed significant positive correlations with biomarkers (*p* < 0.05), most were not among the top 20 most abundant genera (Figure S2). This further indicates that *Staphylococcus* and *Mammaliicoccus* play critical roles in mediating GR14’s alleviation of inflammatory responses in model mice. In summary, these results indicate that the intervention with GR14 can significantly improve the changes in skin microbiota caused by model establishment, allowing the skin microbiota structure to gradually return to a normal state.

## 4. Conclusions

In this study, the effects of a novel yak bone collagen-derived anti-inflammatory peptide GR14 were investigated. The intervention of GR14 alleviated the inflammatory response of mice under DNCB stimulation. It is noteworthy that the high-dose GR14 intervention exhibited nearly equivalent efficacy to the dexamethasone-treated group, while avoiding side effects such as dexamethasone-induced reduction in skin moisture, demonstrating promising application prospects. Mechanism of action analysis indicated that GR14 might attenuate the inflammation of mice by inhibiting the NF-κB signaling pathway and modulating the skin microbiota. However, restricted by the limitations of sequencing methods, this study only investigated the microbial-mediated effects of GR14 on improving model mice at the genus level, and the metabolites in the serum or intestine were not detected. Future studies will employ integrated analysis approaches combining metagenomics and metabolomics to conduct in-depth investigations into the mechanisms by which GR14 alleviates skin inflammation through skin microbiota regulation.

## Figures and Tables

**Table 1:** The alpha diversity of skin microbiota in each group.

Group	Ace	Chao	Shannon	Simpson
NC	301.64 ± 66.01	295.38 ± 62.83	3.44 ± 0.24	0.82 ± 0.01
MC	1022.96 ± 561.72	1009.96 ± 554.68	7.72 ± 0.18 *	0.97 ± 0.01 *
PC	126.59 ± 50.81	125.52 ± 50.59	2.70 ± 0.16 *	0.75 ± 0.02 *
LD	838.78 ± 351.40	832.82 ± 346.17	5.51 ± 0.30 *	0.91 ± 0.01
MD	470.37 ± 61.22	468.00 ± 60.14	3.94 ± 0.21	0.83 ± 0.02
HD	200.84 ± 45.12	199.27 ± 44.77	2.79 ± 0.18	0.75 ± 0.01 *

Note: NC, blank control group; MC, model group; PC, dexamethasone acetate treatment group; LD, low-dose GR14 group; MD, medium-dose GR14 group; HD, high-dose GR14 group. n = 4 per group. “*” indicating the group has a significant difference with the NC group, and the *p*-value below 0.05.

## Data Availability

The original contributions presented in the study are included in the article/Supplementary Material, further inquiries can be directed to the corresponding author.

## Supplementary Materials

The following supporting information can be downloaded at: https://www.mdpi.com/article/10.3390/foods14244238/s1.
