# Supplementary material for: Novel Anti-Inflammatory Bioactive Peptide Derived from Yak Bone Collagen Alleviates the Skin Inflammation of Mice by Inhibiting the NF-κB Signaling Pathway and Modulating Skin Microbiota
Source: Foods. 2025 Dec 10;14(24):4238. doi: 10.3390/foods14244238 (PMC12732001; doi:10.3390/foods14244238)
Supplement: Supplementary file 1 [file foods-14-04238-s001.zip › foods-3980833-supplementary.pdf]

Novel anti-inflammatory bioactive peptide derived from yak bone collagen alleviates the skin inflammation of mice by inhibiting the NF-κB signaling pathway and modulating skin microbiota

Zitao Guo[1], Tao Shi[1], Pengfei Xu[2], Zijun Wang[2], Bo Hu[3], Yu Xin[2], Zhongpeng Guo[2], Zhenghua Gu[2], Dake Dong[4], and Liang Zhang [2, 5]*

[1] School of Food and Biological Engineering, Jiangsu University, Zhenjiang, China

[2] National Engineering Research Center of Cereal Fermentation and Food Biomanufacturing, School of Biotechnology, Jiangnan University, Wuxi, China

[3] National Engineering Research Center for Functional Food, Jiangnan University, Wuxi, China

[4] Department of Dermatology, Affiliated Hospital of Jiangnan University, Wuxi 214122, China

[5] Engineering Research Center of the Ministry of Education for Wound Repair Technology, Jiangnan University, Affiliated Hospital of Jiangnan University, Wuxi, 214000, China.

*** Corresponding authors:**

Liang Zhang

zhangl@jiangnan.edu.cn

1. The original gel of western blotting

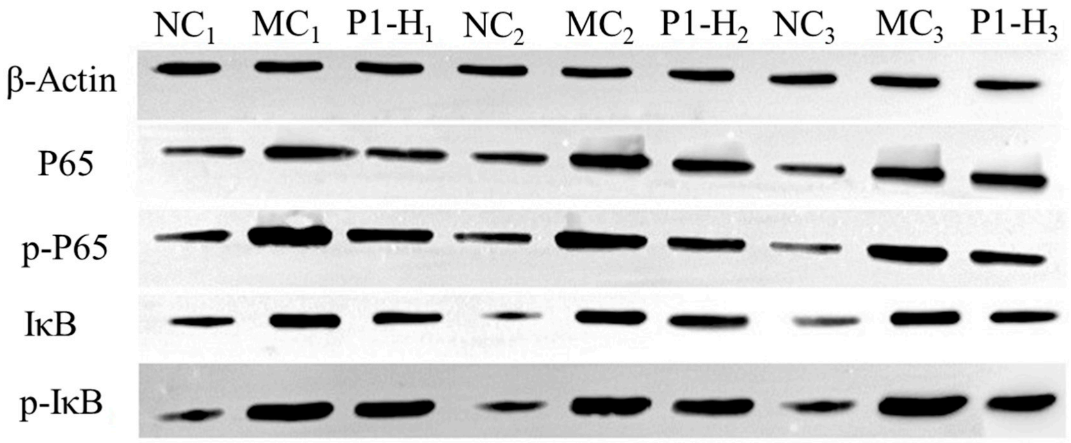

Figure S1 The original gel of western blotting. NC: blank control group; MC: model group; P1-H: high dose P1 group. The lowercase 1-3 corresponding three samples in each group. p-P65: phosphorylation P65; p- IκB: phosphorylation IκB.

2. The skin microbiota of each group

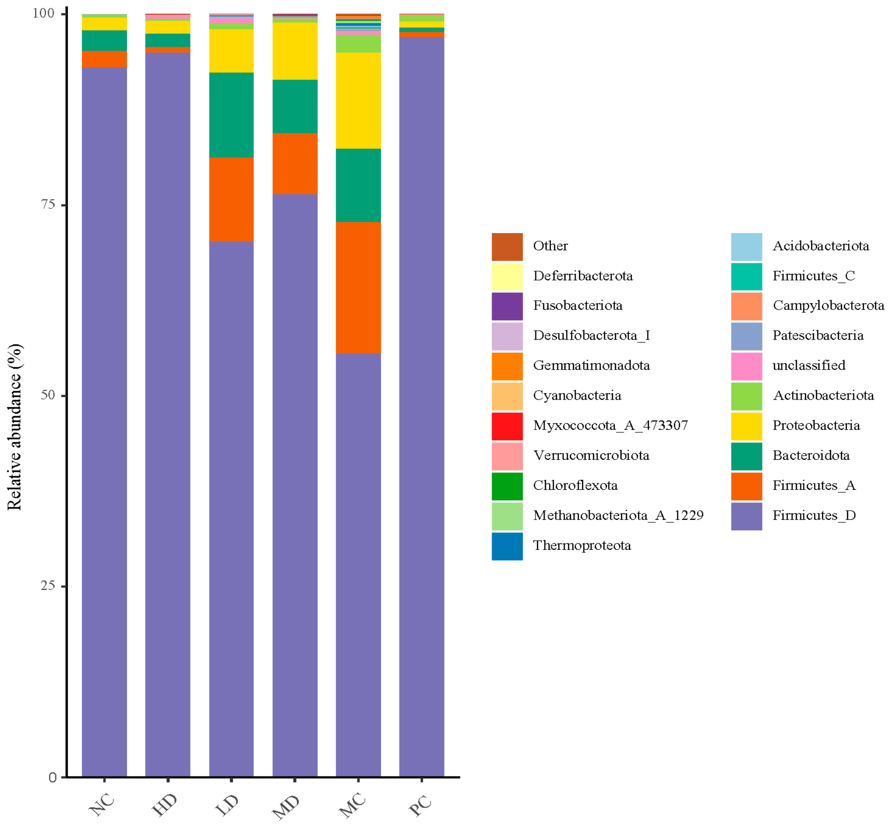

Figure S2 The composition of skin microbiota at the phylum level. NC: blank control group; MC: model group; PC: positive control group; LD: low dose group; MD: middle dose group; HD: high dose group.

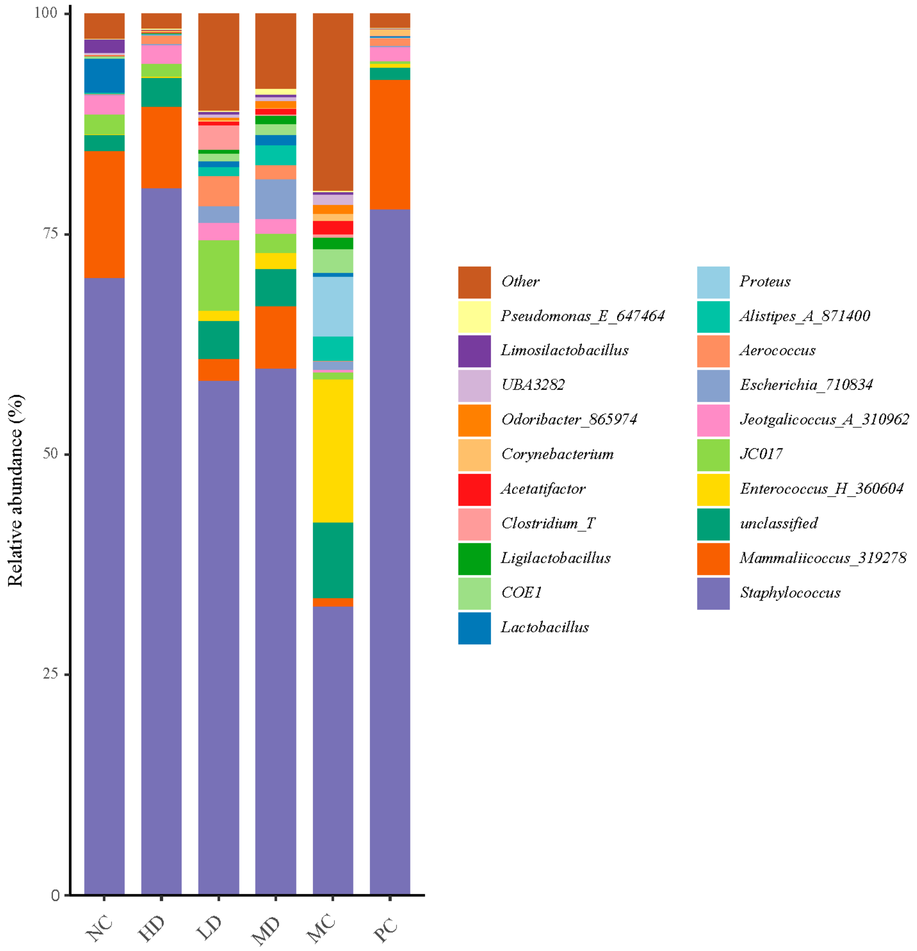

Figure S3 The composition of skin microbiota at the genus level. NC: blank control group; MC: model group; PC: positive control group; LD: low dose group; MD: middle dose group; HD: high dose group.
